# The Development and Validation of the Spiritual Values Scale for Primary School Children in the Turkish Context

**DOI:** 10.1007/s10943-025-02378-4

**Published:** 2025-07-04

**Authors:** Erdal Zengin

**Affiliations:** Department of Elementary Education, Faculty of Education, Fırat University, Elazığ, Turkey

**Keywords:** Spiritual values, Primary school children, Scale validation, Turkey, Spiritual resilience, Child development

## Abstract

The present study was conducted with the objective of developing and validating the Spiritual Values Scale for Primary School Children (SVS-PSC) as a reliable and culturally sensitive instrument to assess spiritual values among Turkish primary school students. The scale development process followed a multi-phase psychometric framework, including the generation of an item pool, expert review, exploratory factor analysis (EFA), confirmatory factor analysis (CFA), and criterion-related validation. A study was conducted with 400 children aged 9–11 in order to ascertain the structure of the EFA. The results of the study revealed a unidimensional structure which explained 47.716% of the total variance, with item loadings ranging from .512 to .690. The KMO value (.806) and Bartlett’s test (χ^2^ = 613.902, *p* < .001) indicated the suitability of the data for factor analysis. The CFA, based on a sample of 260 children, supported the one-factor model with acceptable fit indices (χ^2^/df = 2.231; RMSEA = .080; CFI = .914; GFI = .930). The criterion validity of the scale was examined through administration of the Spiritual Robustness Scale to a sample of 39 children. A significant positive correlation (r = .371, *p* < .001) was identified, suggesting that the scale effectively captures aspects of spiritual resilience and internal strength. The SVS-PSC demonstrated high reliability (Cronbach’s α = .831), and item-total correlations ranged from .396 to .588. The findings of this study indicate that the SVS-PSC is a psychometrically robust tool for evaluating core spiritual values such as honesty, gratitude, empathy, and responsibility in children. The scale provides researchers and educators with a valid instrument with which to understand spiritual development and foster character education in primary school settings.

## Introductıon

### Problem and Importance of the Research

Spiritual values are among the fundamental principles that guide an individual's personal development, shape their moral decisions, and enable them to establish healthy relationships with society (Akram et al., 2021; Roseth, 2016; Thornberg & Oğuz, 2016). It is evident that values such as respect, compassion, honesty, and responsibility have the capacity to facilitate the assumption of active, constructive roles within society, thereby reinforcing social cohesion (Schwartz, 2012; Yao & Wong, 2020). The fact that these values are usually internalised during childhood makes this developmental stage the most critical step in value-based character building (Haslip & Gullo, 2018; Betawi, 2018; Kızılgeçit et al., 2024). It is particularly noteworthy that the school environment provides a significant context that influences children's social and moral orientations. Teachers, in their capacity as role models, play a pivotal role in this process (Ailwood et al., 2011; Barahate, 2014).

Recent studies have indicated that the moral content in textbooks across various countries is predominantly symbolic, with values being incorporated only superficially (Imelwaty et al., 2022; Tan et al., 2018; Rezaei et al., 2021). In particular, individual-level values such as benevolence, kindness and tolerance are prioritised, but contextual and cultural diversity are often ignored (Puspitasari et al., 2021; Sulistiyo et al., 2020). This poses a significant challenge for educators seeking to promote children's character development in a multifaceted manner. It is evident that education systems should adopt a holistic approach to value education that is appropriate to the developmental needs of children.

When evaluated in the context of Turkey, the absence of a scale of spiritual values for primary school students that is appropriate for age development, takes into account the cultural context, and has been tested for psychometric validity and reliability is a notable deficiency in the literature (Ahmad et al., 2014; UNESCO, 2005). Existing studies have predominantly concentrated on religiously referenced or adult-based assessments, a strategy that is inadequate in addressing the distinctive requirements of primary school children. The objective of this study is to develop a valid and reliable measurement tool to assess the spiritual value levels of primary school children. It is widely accepted that childhood represents the most fundamental stage in the development of an individual's ethical sensitivity, social responsibility, awareness and personality. The absence of measurement tools appropriate for this age group represents a significant lacuna in both educational practices and scientific research. Consequently, the scale to be developed will facilitate teachers' structuring of classroom observations, shaping of educational policies based on data, and more systematic support for children's value-based development.

### Purpose of the Research

The objective of this research is to develop an assessment tool that can validly and reliably measure the spiritual value levels of primary school children in the Turkish context. Spiritual values have been shown to play a significant role in both personal development and adaptation to social life. The period of childhood is widely regarded as the most critical developmental stage in terms of the internalisation of these values. It is particularly noteworthy that the school environment is the first social structure in which children are systematically introduced to the concept of values education. However, the absence of a comprehensive scale of spiritual values for primary school children in Turkey, grounded in scientific foundations and tailored to the developmental characteristics of young learners, is identified as a significant lacuna. The measurement tool to be developed in this direction aims to provide both educators and researchers with a scientific and functional tool that can be used to determine the spiritual development levels of children.

## Lıterature Revıew

### Spiritual Values and Child Development

Spiritual values include basic moral principles such as love, respect, compassion, truthfulness, responsibility and sharing that regulate both the inner world and social relations of the individual (Arthur, 2019; DfE, 2019; Ekşi & Okan, 2021). These values are decisive in the development of children's critical social skills such as empathy towards others, pursuing justice and fairness in their decisions, and living in harmony with society (Ekşi et al., 2021; Suissa, 2015; Jerome & Kisby, 2019). Especially in pluralistic society structures, tolerance towards differences requires supporting the spiritual aspects of individuals from an early age (Hurley and D’Arcy, 2023). In this context, ethical sensitivity and meaningful social interactions should be provided in educational environments for children to show not only academic success but also value-based development (Chan, 2024). Because moral values adopted at an early age form the basis of children's lifelong personal and social attitudes (Cassam, 2019; OfSTED, 2019).

Childhood is the most critical developmental stage in which an individual's character and value world are shaped (Reimer et al., 2022). Value education given in this period supports children to acquire skills that will contribute to social life such as taking responsibility, helping and acting fairly (Ahluwalia-McMeddes et al., 2024; Jerome & Kisby, 2019). Especially in the programmes implemented in educational environments, values should be concretised not as abstract concepts, but by associating them with situations that students encounter in their daily lives (Nan et al., 2022; Jassemi, 2023). At this point, the role of teachers should be considered not only as a transmitter of information, but also as a guide who supports the spiritual development of children (Busher et al., 2020; Revell & Bryan, 2018). The values internalised by the child expand the scope of value education by directly affecting not only individual orientations but also peer relations, in-group interactions and sense of social responsibility (Cassam, 2019; Figueira et al., 2025).

The relationship between spiritual values and character education presents a reciprocal structure that deeply affects children's social-emotional development processes (Jassemi, 2023; Nan et al., 2022). Individual character qualities such as self-control, perseverance, patience, and determination become more permanent and meaningful only when they are supported by spiritual values such as empathy, benevolence, and consideration for the rights of others (Arthur, 2019). It is seen that certain values have a guiding effect on children's ethical behaviour and decision-making processes; especially value-oriented attitudes such as loyalty, purity and justice are understood to shape social interactions (Haidt, 2012; Reimer et al., 2022). Strengthening the relationship between character education and spiritual development in education systems ensures that children are oriented towards not only individual but also social success (Ho & Barton, 2020; Jerome & Kisby, 2019). Therefore, processing values at both cognitive and emotional levels contributes to the development of children as consistent, balanced and sensitive individuals (Ahluwalia-McMeddes et al., 2024). **In this context, holistic approaches to child development should not ignore the complementary nature of spiritual values and character education.**

## Researches on the Measurement of Spiritual Values

In the international literature, research on the measurement of moral values is mostly based on adolescent and adult individuals. For example, the Adolescents' Value Scale developed by Chen (2008) provides an important start in conceptualising moral values, while the Virtue Adjectives Rating Scale developed by Mu and Gu (2010) evaluated the virtue levels of individuals in a multidimensional way. Similarly, Wang (2008) tried to distinguish between emotional and ethical orientations of an individual by measuring gratitude, and Girard and Mullet (1997) were able to describe abstract values such as forgiveness. However, all of these scales are either limited to older samples or contextually based on Western culture. In this context, the studies conducted by Francis et al. (2014) in the UK context focused on specific sub-areas such as racism, illegal behaviours and sexual morality; however, this approach is far from reflecting the value world of children in a holistic manner. Therefore, it is seen that foreign scales are not directly applicable to cover the spiritual development characteristics of primary school children.

The scales developed in Turkey generally focus on religious orientations or young adults. For example, the Spiritual Transcendence Scale (İme et al., 2019) was developed to measure the spiritual orientations of university students, while the Turkish adaptations of the Spiritual Meaning Scale and Spirituality Expressions Inventory (Şahin et al., 2017) analysed more cognitive and emotional dimensions of spirituality. The Spiritual-Humanitarian Values Tendency Scale (Güneş, 2015a, 2015b) measures factors such as spiritual values, respect and love in adult individuals. While the scale developed by Koç and Budak (2021) evaluates the effect of social activities on values education, it does not have the capacity to directly measure the spiritual content specific to children. The common point of these scales is that they either rely too much on religious references or neglect the developmental characteristics of childhood. These deficiencies reveal the need for scales specially prepared for children, shaped on a secular level and taking into account the cultural context.

The new scale approach developed in line with this need aims to assess the moral values of primary school children in a holistic, developmental and culturally based manner. Items adapted from Frost et al.'s (1990) Multidimensional Perfectionism Scale (FMPS) (McArdle, 2010; Yang, 2007; Zi & Zhou, 2006) to measure an individual's personal moral standards and concern about making moral mistakes have the potential to directly assess children's spiritual sensitivity. At the same time, the contents of instruments such as Wei and Hwang's (1998) misbehaviour scale can be used to analyse children's cognitive and emotional responses to normative values. In this context, the proposed scale aims to measure children's spiritual development in a multidimensional way by addressing values such as empathy, compassion, responsibility and inner peace from both cognitive and behavioural perspectives. Such a scale will provide a scientific basis that will contribute not only to educational policies but also to psychological counselling and social adaptation strategies.

## Method

### Research Design and Purpose

This study was designed as a quantitative research based on a scale development method to measure the spiritual values of primary school students in the Turkish context. The scale development process involves the creation of a new measurement tool to assess the spiritual values of primary school children and the validity and reliability analyses of this tool (Byrne, 2016; Clark & Watson, 1995; Okan & Şahin, 2024; Worthington & Whittaker, 2006).

The main purpose of this study is to develop the *Spiritual Values Scale for Primary School Children (MDS-SC*), a valid and reliable measurement tool that can evaluate the spiritual values of primary school children in a holistic way. The scale aims to objectively and scientifically measure the dimensions of spiritual values such as love, respect, compassion, responsibility, truthfulness that children exhibit in their daily lives. In this context, the research process was completed in three stages.

### Participants

This research was conducted based on three different data sets in the process of developing the Spiritual Values Scale for Primary School Children. At each stage of the scale development process, the demographic characteristics of the participants (such as age, gender, grade level) were examined and their distribution according to these variables was evaluated.

### Exploratory Factor Analysis (EFA)

In the first stage, data were collected from 400 primary school students to determine the factor structure of the scale. Of the participants, 170 were girls (42.5%) and 230 were boys (57.5%). These students, aged between 9 and 11 years, were selected from different grade levels (3rd and 4th grade). At this stage, the sub-dimensions and factor loadings of the scale were determined.

### Confirmatory Factor Analysis (CFA)

In the second stage, data were collected from 260 students in order to verify the determined structure of the scale. This group included 159 female (61.2%) and 101 male (38.8%) students. CFA results showed that the proposed factor structure of the scale was valid and the model fit indices (CFI, TLI, RMSEA) were acceptable.

### Criterion Validity

In the third stage, data were collected from 39 students for criterion validity analyses. This data set was used to examine the relationships between levels of spiritual values and measures such as positive character traits, prosocial behaviours or self-perception. In the criterion validity studies, correlation analyses were conducted with scales that measure similar psychological constructs developed for children. The positive and significant correlations obtained support the validity of the developed scale.

The Spiritual Values Scale for Primary School Children developed in this study was designed to understand, evaluate and develop children's spiritual values. The scale was structured to measure the extent to which children adopt basic spiritual values such as honesty, benevolence, gratitude, patience, and responsibility. Criterion validity studies conducted within the scope of validity analyses revealed that spiritual values were significantly related to children's spiritual robustness (Okan & Ekşi, 2025). These results show that the scale in question is a reliable and valid measurement tool for both scientific research and practitioners.

### Procedure

The following section summarises the development process of the Spiritual Values Scale for Primary School Children (MDS-SC) in the Turkish context.

### Theoretical Foundation

The Spiritual Values Scale for Primary School Children (MDS-SC) was developed to assess the basic spiritual values (love, respect, responsibility, truthfulness, helpfulness, compassion, etc.) that children exhibit in daily life. Spiritual values have an important place in making sense of both the individual's inner world and the relationships he/she establishes with his/her environment (Haydon, 2006; Lickona, 1991).

In the literature, it is stated that spiritual values have positive effects on children's social adaptation, prosocial behaviours and character development (Berkowitz & Bier, 2014; Nucci & Narvaez, 2008). In this context, the developed MTS-IW aims to measure the spiritual value levels of primary school children and to contribute to academic research in this field.

### Item Pool Development

The item pool for the Spiritual Values Scale was developed through a comprehensive review of relevant literature and interviews with experts in psychological counseling, education, and educational measurement. Core spiritual values such as love, respect, compassion, responsibility, honesty, and solidarity formed the thematic foundation of the scale. Drawing on existing scales and educational programs (Lickona, 1991; Berkowitz & Bier, 2014), 35 preliminary items appropriate for children's developmental stages were initially formulated. These items were then evaluated for content validity by five academic experts, and, based on their feedback, several items were revised, simplified, or eliminated. As a result of this expert review process, 28 refined items were finalized for the pilot application.

### Expert Review and Content Validity

The expert panel formed to ensure content validity evaluated the comprehensibility of the items, the appropriateness of the scope and the appropriateness of the items to children's developmental characteristics. In line with the expert opinions, some of the statements were made more suitable for children's daily life and unnecessary repetitions were removed. At the end of this process, 21 items were agreed upon.

#### Pilot Application

The scale was applied to a pilot sample of 40 primary school students (3rd and 4th grade). The ages of the participants ranged between 8 and 11 years. In the pilot study, necessary adjustments were made to make some words and expressions more easily understood by children. After the pilot application, the final version of the scale was determined as 21 items.

#### Data Collection Process

The data were collected from primary school students in public schools in Elazığ and Tunceli provinces through face-to-face applications. Participants were included in the study with the approval of their teachers and families. The purpose of the study was first explained to the students and then the Spiritual Values Scale was applied. Confidentiality and voluntariness principles were observed during the data collection process.

#### Validity and Reliability Analyses

Exploratory Factor Analysis (EFA) and Confirmatory Factor Analysis (CFA) were conducted to evaluate the construct validity of the scale. According to the EFA results, a single factor representing spiritual values emerged:

The items with low factor loadings were removed from the scale and the final scale consisting of 11 items was obtained. CFA results revealed that the model showed an acceptable fit (e.g., RMSEA < 0.08, CFI > 0.90).

For content validity, the content validity ratios calculated in line with expert opinions using Lawshe’s (1^975^) method were found to be at an acceptable level. Within the scope of criterion validity, positively significant relationships were obtained between MDS-IW and prosocial behaviour scale and moral sensitivity scale.

In the reliability analyses, the Cronbach's alpha coefficient of the scale was found to be α = 0.87, which indicates high internal consistency. In addition, item-total correlations were found to be above 0.60.

#### Data Analysis

In the first stage, the scale items were prepared in line with the relevant literature and theoretical framework, and necessary adjustments were made by taking expert opinions. Then, Exploratory Factor Analysis (EFA) was applied to determine the factor structure of the scale (Fabrigar et al., 1999; Okan & Ekşi, 2025; Tabachnick & Fidell, 2019). As a result of this analysis, the factor structure representing children's spiritual values was revealed.

#### Confirmatory Factor Analysis (CFA)

In the second stage, Confirmatory Factor Analysis (CFA) was applied to test the accuracy of the factor structure obtained by EFA. CFA enabled the factor structure of the developed scale to be confirmed by model fit indices (Brown, 2015; Kline, 2016; Okan & Okan, 2024).

#### Criterion Validity and Reliability Analyses

In the third stage, criterion validity analyses were conducted to examine the relationship between the scale and valid measurement tools. The relationships between spiritual values and variables such as prosocial behaviour, empathy and moral sensitivity were examined. In addition, the internal consistency of the scale was evaluated by calculating Cronbach's alpha and composite reliability coefficients (Nunnally & Bernstein, 1994; Hair et al., 2020). The test–retest reliability of the scale was tested with two-week intervals.

These stages are of critical importance in terms of ensuring both validity and reliability criteria of the scale and demonstrating the usability of the *Spiritual Values Scale for Primary School Children* as a scientific tool for measuring children's spiritual value levels.

## Fındıngs

### Findings Related to Scale Development

In this section, the development process of the *Spiritual Values Scale for Primary School Children (MDS-SC*) and the statistical findings obtained are discussed in detail. The steps followed in the development process of the scale and the results of the analyses obtained in this process are presented in a structured manner.

### Validity

The validity and reliability of a scale is one of the basic criteria that determine its usability in scientific research. In this study, the validity of the MDS-IW was evaluated with various statistical analyses. Validity is related to the ability of the scale to measure the concept (spiritual values) that it wants to measure in a purposeful and accurate way.

As a result of the validity analyses of this scale, which was developed to determine the spiritual value levels of primary school children, findings supporting both its construct validity and its suitability for the purpose of measurement were obtained.

In this context, the validity of the scale:

- Determination of factor structure by Exploratory Factor Analysis (EFA),
- Testing this structure with Confirmatory Factor Analysis (CFA),
- Content validity based on expert opinions,
- Criterion validity through correlation analyses with related scales
- methods were evaluated.

### Exploratory Factor Analysis (EFA) Findings

According to the EFA results, *the Spiritual Values Scale for Primary School Children (MDS-SC*) has a one-factor structure. The single factor obtained as a result of the analysis explained 54.871% of the total variance of the scale. In the social sciences literature, a total variance explained between 40 and 60% is considered sufficient for scale development studies (Hair et al., 2020; Tabachnick & Fidell, 2019).

In addition, the factor loadings of the items were found to be 0.50 and above; some items with low factor loadings were removed from the scale. As a result of this process, the scale reached its final form consisting of 21 items. These findings show that the scale has sufficient construct validity in measuring the spiritual values of primary school children.

The results of the factor analyses revealed that *the Spiritual Values Scale for Primary School Children (MDS-SC*) had a single-factor structure and that this structure was able to evaluate children's spiritual values holistically.

The one-factor structure of the scale shows that basic spiritual values such as love, respect, compassion, responsibility, truthfulness and benevolence are effective on children's attitudes and behaviours as a whole and that these values can be evaluated together.

As a result of the Exploratory Factor Analysis (EFA), the factor explaining 47.716% of the total variance reveals that the scale has sufficient construct validity (Hair et al., 2020; Tabachnick & Fidell, 2019).

These findings confirm that the *Spiritual Values Scale for Primary School Children* is a reliable and valid measurement tool for measuring children's spiritual values and can be used in scientific research Tables 1 and 2.

**Table 1:** Distribution of Participants by Type of Analysis and Gender

Type of Analysis	Total (n)	Female Students (n)	Male Student (n)	Girl %	Male %
Exploratory Factor Analysis (EFA)	400	170	230	42,5%	57,5%
Confirmatory Factor Analysis (CFA)	260	159	101	61,2%	38,8%
Criterion Validity	39	20	19	51,3%	48,7%
Total	**699**	**349**	**350**	**49,9%**	**50,1%**

Bold values indicate the total number of participants, emphasizing the overall sample size

**Table 2:** Variance Explained for the Spiritual Values Scale as a Result of EFA

Factor	Total Explained Variance
Spiritual Values Scale	%47,71
Total	%47,71

When Table 3 is examined, it is seen that the results of KMO and Bartlett's Test for *the Spiritual Values Scale for Primary School Children (MDS-SC*) show that the data set is suitable for factor analysis.

**Table 3:** KMO and Bartlett's Test Values

Kaiser–Meyer–Olkin Sampling Adequacy		.81
Bartlett's Test of Sphericity	Chi-square Value	613.90
	S. Degree	55
	P	,000

Kaiser–Meyer–Olkin (KMO) Sampling Adequacy value was calculated as 0.81. This value is evaluated at a very good level according to the criteria accepted in the literature (0.90 and above is excellent; 0.80–0.90 is very good; 0.70–0.80 is good; 0.60–0.70 is moderate; below 0.60 is insufficient) and shows the suitability of the data set for factor analysis (Field, 2018).

According to Bartlett's Test of Sphericity results, Chi-square value was found as 613.90, degree of freedom as 55 and significance level as *p* < 0.000. This result shows that there are significant correlations between the scale items at a sufficient level and that the data have suitable properties for factor analysis.

In line with these findings, it was concluded that the data set had sufficient sample size and appropriate data characteristics for the determination of the factor structure of the *Spiritual Values Scale for Primary School Children (MDS-SC*). This reveals that the validity studies of the scale can be carried out in a healthy way.

Scree Plot graph is a visual analysis tool used to evaluate the factor structure of the scale. The graph contributes to the determination of the number of factors based on the eigenvalues of the factors.

When the scree plot graph created for *the Spiritual Values Scale for Primary School Children (MDS-SCF*) is analysed, it is seen that there is a clear and obvious elbow point on the graph. It is noteworthy that there is a sharp decline on the graph especially after the first factor and the slope decreases significantly. From this point onwards, the graph line flattens and the eigenvalues of the factors are below 1.

This clearly reveals that the scale has a single-factor structure. The first factor has the highest eigenvalue and largely represents the dimension of *spiritual values*, which is the basic concept that the scale wants to measure. According to the scree plot graph, the eigenvalues of the other factors following the first factor are below 1 and it is seen that these components do not make a significant contribution to the structure of the scale.

This breakpoint and the change in slope seen in the graph strongly confirm that the *Spiritual Values Scale for Primary School Children (MDS-SC*) is a unidimensional scale. The low eigenvalues of the other factors indicate that all items of the scale measure the same main concept, namely *spiritual values,* in a holistic manner.

These findings, when evaluated together with the results of the exploratory factor analysis (EFA), reveal that the construct validity of the scale is strong and sufficient.

When Table 4 is analysed, it is seen that the factor loadings of the items belonging to the *Spiritual Values Scale for Primary School Children (MDS-SC*) vary between 0.51 and 0.69. Factor loadings show the relationship and representation power of each item with the main concept (spiritual values) that it wants to measure. In the literature, factor loading values of 0.40 and above (Hair et al., 2020) indicate that the relevant item adequately represents the factor; the fact that the loading values obtained in this study are well above 0.50 supports that the construct validity of the scale is strong.

**Table 4:** Load Values of Spiritual Values Scale Items

Articles	Spiritual Values
SV16	,69
SV20	,67
SV27	,66
SV14	,65
SV18	,63
SV25	,63
SV30	,56
SV6	,56
SV11	,56
SV13	,56
SV9	,51

The fact that the factor loadings of the items are high and close to each other shows that the scale is designed in conceptual integrity and all items are effective in measuring children's spiritual value levels. These results reveal that the single-factor structure of the *Spiritual Values Scale for Primary School Children (MDS-SC*) is robust and consistent, and the items can evaluate the concept of spiritual value in a holistic way. The fact that the items have high factor loadings at this level shows that the validity and factor structure of the scale are compatible with the theoretical foundations and that the scale can be used safely in scientific research.

### Confirmatory Factor Analysis

**Fig. 1:**
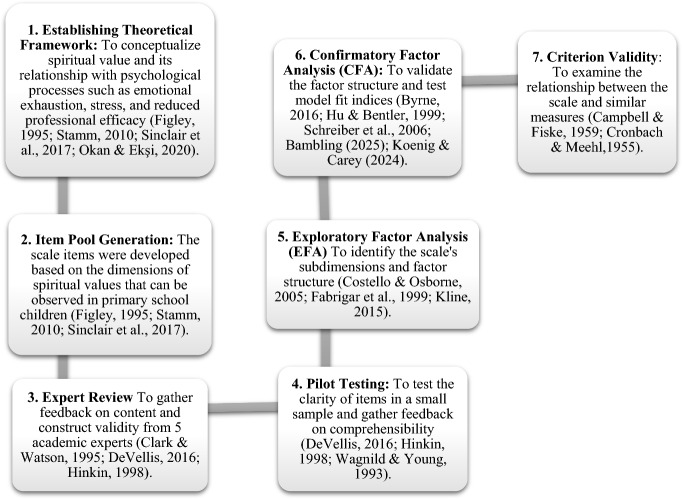
Steps in the Development of the Spiritual Value Scale (Field, 2018; Koenig & Al Zaben, 2021; Koenig & Carey, 2025; Bambling, 2025).

**Fig. 2:**
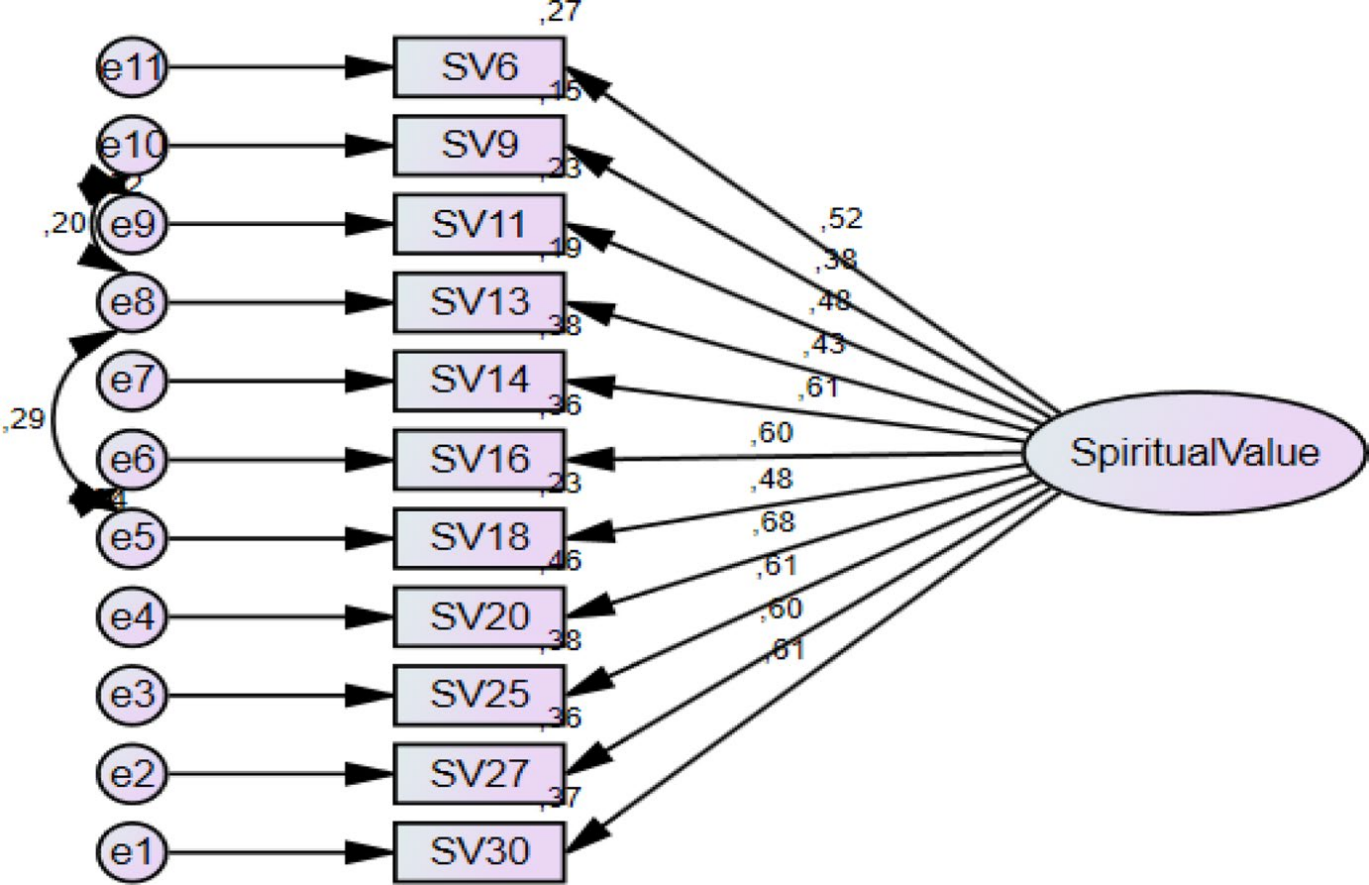
Scree Plot Graph for the Scale

**Fig. 3:**
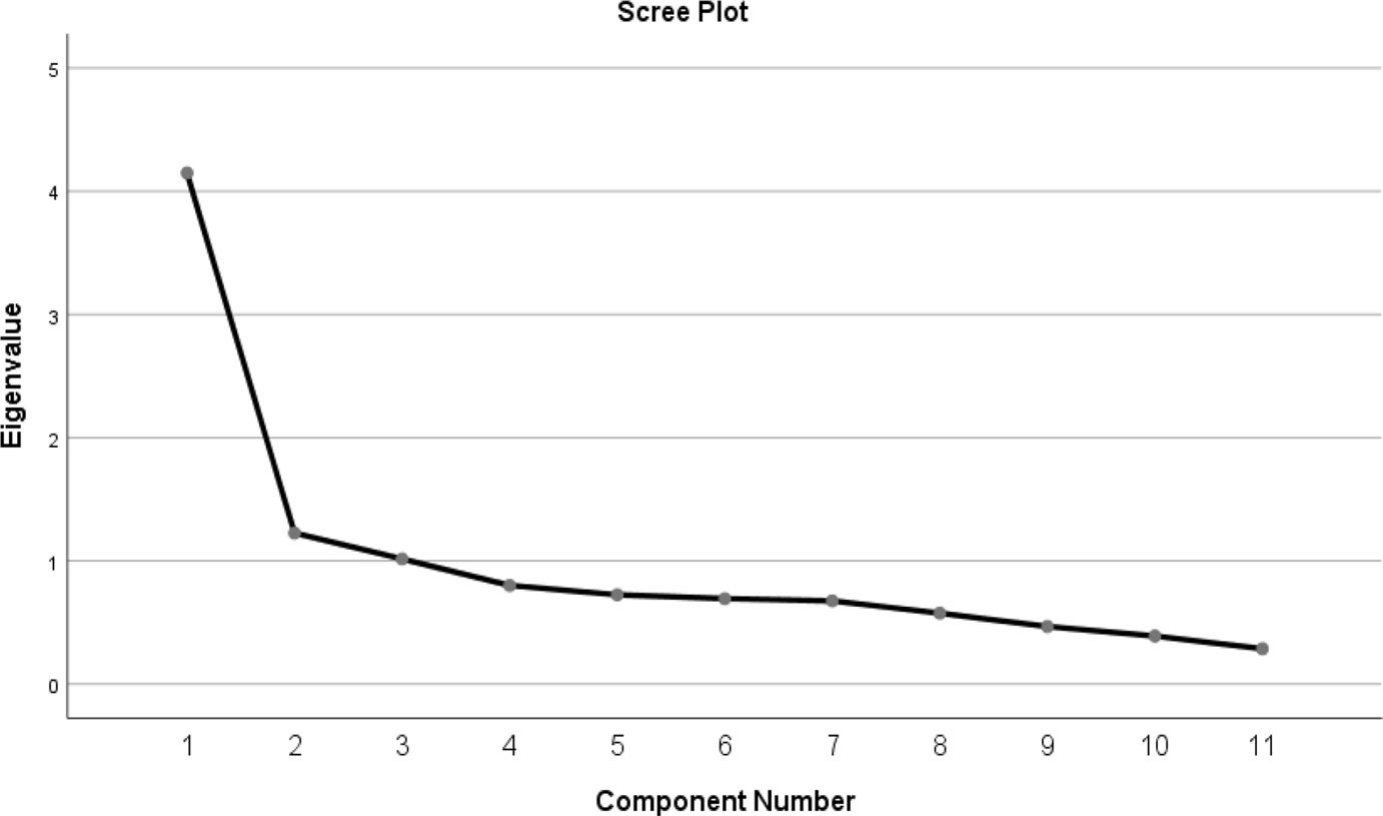
Path Diagram of Confirmatory Factor Analysis for Spiritual Values in Children Scale

The confirmatory factor analysis (CFA) path diagram presented in Figs. 1, 2 and 3 reveals the unidimensional structure of the *Spiritual Values Scale for Primary School Children (MDS-SC*) and the relationships between the items and this dimension in detail.

The factor loadings in the path diagram show the extent to which the scale items represent the dimension of spiritual values. According to the results of the analyses, factor loadings ranged between,52 and,81. This finding shows that the scale items are generally strongly related to the spiritual values construct.

Especially SV27 (,81) and SV18 (,68) stand out as the items that most strongly represent the conceptual structure of the scale. However, the items with lower factor loadings such as SV6 (,52) and SV9 (,38) are also at acceptable levels and make a significant contribution to the overall structure of the scale.

In the literature, factor loadings of 0.40 and above are considered as acceptable limits (Hair et al., 2020). In this context, the fact that the factor loadings of all items are 0.40 and above confirms that the scale items show a theoretically consistent and significant relationship with the dimension of spiritual values.

The CFA findings strongly support that the *Spiritual Values Scale for Primary School Children (MDS-SCF*) was developed in a unidimensional structure and can measure spiritual values in a reliable and valid way.

The fact that the majority of the items have high factor loadings reveals that the scale was prepared in conceptual integrity and is an effective tool for measuring the spiritual value levels of primary school children.

When Table 5 is examined, it is seen that the model obtained in line with the results of confirmatory factor analysis (CFA) of the *Spiritual Values Scale for Primary School Children (MDS-SC*) generally shows an acceptable and good fit.

**Table 5:** Comparison of Standard Goodness of Fit Criteria and Research Results

Fit Dimensions	Good Fit	Acceptable Compliance	Concordance Values Obtained in the Study
c2/df	0 ≤ c2/df ≤ 2	2 ≤ c2/df ≤ 3	2.23
RMSEA	0 ≤ RMSEA ≤ 0.05	0.05 ≤ RMSEA ≤ 0.08	0.08
SRMR	0 ≤ SRMR ≤ 0.05	0.05 ≤ SRMR ≤ 0.10	0.02
IFI	0.95 ≤ NFI ≤ 1.00	0.90 ≤ NFI ≤ 0.95	0.92
CFI	0.95 ≤ CFI ≤ 1.00	0.90 ≤ CFI ≤ 0.95	0.91
GFI	0.90 ≤ GFI ≤ 1.00	0.85 ≤ GFI ≤ 0.90	0.93
TLI	0.90 < RFI < 1.00	0.85 < RFI < 0.90	0.88

The χ^2^/df value was found to be 2.231 and is in the range of 2 ≤ χ^2^/df ≤ 3, indicating that the model has an acceptable fit. The RMSEA value was determined as 0.080 and it is in the range of 0.05 ≤ RMSEA ≤ 0.08, indicating that the model has an acceptable fit. Since the SRMR value is below 0.05 with 0.016, it indicates that the model has a very good fit. The IFI (0.916), CFI (0.914) and GFI (0.930) values are between 0.90 and 0.95, indicating that the model provides an acceptable fit. TLI value was calculated as 0.878 and it is between 0.85 and 0.90, which shows that the model has an acceptable fit.

The confirmatory factor analysis (CFA) findings strongly support the validity and reliability of the single-factor structure of the *Spiritual Values Scale for Primary School Children (MDS-SC*). In particular, SRMR and GFI values are at very good levels, indicating that the model fits the data very well. The fact that all other fit indices are within acceptable limits shows that the scale was developed in accordance with the conceptual integrity and theoretical framework.

In conclusion, the CFA results show that the unidimensional structure of the *Spiritual Values Scale for Primary School Children (MDS-SC*) is valid and the scale works consistently with the data. These findings confirm that the scale is a valid and reliable measurement tool that can be used safely in scientific research to measure the spiritual values of primary school children.

### Reliability Findings

#### Criterion Validity of the Scale

In order to determine the criterion validity of the Spiritual Values Scale for Primary School Children, a comparative study was conducted with the Spiritual Robustness Scale, which assesses the spiritual resilience levels of children. Since both scales assess individuals' spiritual orientations, inner power sources and search for meaning, it is important to examine the relationship between them in terms of criterion validity.

When Table 6 is examined, it is seen that there is a positive and significant relationship between the Spiritual Values Scale for Primary School Children and Spiritual Robustness (*r* = *0.37, p* < *0.001*). This finding shows that as children's spiritual values level increases, their capacity to cope with the difficulties of life, to produce meaning and to use their inner power resources (spiritual resilience) also increases. In addition, the spiritual resilience scale has a high level of internal consistency (*r* = *0.71*), which supports that the construct is measured reliably. These findings indicate that the Spiritual Values Scale has criterion validity and establishes consistent relationships with similar constructs (e.g., spiritual resilience). Therefore, this scale can be considered as an effective tool in understanding the spiritual dimensions related to children's psychological resilience and well-being.

**Table 6:** The Relationship between Spiritual Values and Spiritual Robustness in Children

Variables	Spiritual Values (r)	Spiritual Robustness (r)
Spiritual Values	1.000	0.37*
Spiritual Robustness	0.37*	0.71

^*^P < ,001

When Tables 7 and 8 is examined, it is seen that the scale has a high level of reliability in line with the internal consistency coefficients and item-total correlation values of the *Spiritual Values Scale for Primary School Children (MDS-SC*). The overall Cronbach's Alpha value of the scale was calculated as,831. This value shows that the scale has a very high internal consistency and is a tool that can reliably measure the spiritual value levels of primary school children.

**Table 7:** Spiritual Values Scale Internal Consistency Coefficients and Item-Total Correlations and Cronbach's Alpha Values

Articles	Corrected Item-Total Correlation	Cronbach's Alpha if Item Deleted
Spiritual Value		0.83
SV16	0.59	0.81
SV20	0.58	0.81
SV27	0.55	0.81
SV14	0.56	0.81
SV18	0.51	0.82
SV25	0.53	0.81
SV30	0.49	0.82
SV6	0.48	0.82
SV11	0.46	0.82
SV13	0.45	0.82
SV9	0.40	0.83

**Table 8:** Test–Retest Reliability Results of the Spiritual Values Scale for Primary School Children

Test Administration	Sample Size (n)	Time Interval	Correlation Coefficient (r)
First Application	35	–	–
Second Application	33	3 weeks	.87

When the item-total correlation values are analysed, it is seen that the items show significant and strong relationships with the total score of the scale. These values vary between 0.396 and 0.588, which are well above the limit values of 0.30 and above accepted in the literature (Nunnally & Bernstein, 1994). This situation reveals that all items of the scale make a significant contribution to the *spiritual values* construct to be measured. Especially items such as SV16 (,588), SV20 (,580), SV27 (,550) and SV14 (,557) stand out as items that strongly represent the general structure of the scale.

In addition, Cronbach's Alpha values obtained when any item was removed from the scale ranged between 0.809 and 0.827. This shows that the removal of any item does not lead to a significant change in the overall reliability of the scale. The fact that the Cronbach's Alpha value changed very little when the items were removed confirms that the items of the scale complement each other and the scale has a holistic structure.

These findings strongly support that the *Spiritual Values Scale for Primary School Children (MDS-SC*) is a reliable measurement tool in terms of internal consistency and that it can robustly measure children's spiritual value levels in accordance with the purpose for which the scale was developed. The fact that both the item-total correlation values of the scale are at a sufficient level and the overall Cronbach's Alpha coefficient is high reveals that the psychometric properties of the scale are strong and scientifically valid.

In order to evaluate the temporal stability of the Spiritual Values Scale for Primary School Children, a test–retest reliability analysis was conducted with 33 students over a three-week interval. Initially, 35 students participated in the first administration; however, two students were unavailable during the second session. The Pearson correlation coefficient between the two administrations was calculated as r = 0.87, indicating a high level of stability and reliability over time.

## Conclusion and Discussion

### Evaluation of Findings

The present study examined the psychometric properties of the Spiritual Values Scale (MDS-SC), which was developed to measure the spiritual value levels of primary school children in the Turkish context. The findings obtained at the conclusion of the scale development process can be summarised as follows:

The scale exhibits a unidimensional structure, embodying fundamental spiritual values such as love, compassion, truthfulness, respect, helpfulness and responsibility through a holistic lens. The results of the EFA and CFA indicated that this structure of the scale was statistically valid.

The internal consistency of the scale was found to be high. The Cronbach's Alpha coefficient was found to be 0.831, and the item-total correlations were observed to exceed the limits that are typically accepted within the relevant literature. These findings indicate the reliability of the scale. The scale items represent the spiritual behaviours that children can exhibit in their daily lives. The material is appropriate for the age group in terms of both conceptual integrity and comprehensibility. The fact that the contents of the item were supported by both expert opinions and pilot applications confirms the suitability of the scale for the target group.

The findings indicate that the developed measurement tool is both valid and reliable, and can effectively measure the spiritual value levels of primary school children.

### Discussion

The findings of the study address a significant gap in the existing literature by demonstrating that the spiritual values of children can be evaluated in a quantifiable and systematic manner. The validity of the single-factor structure demonstrates that children do not perceive spiritual values as separate abstract concepts, but rather as a complementary and intertwined integrity. This perspective aligns with the insights of Lickona (1991) and Arthur (2019), who have posited that character education and value education function in a complementary manner.

In a similar vein, the scale developed by Nazam and Husain (2016) demonstrates that spiritual values are perceived in a multidimensional yet holistic manner across diverse age groups. The structure of the scale encompasses fundamental areas such as altruism, humanism and emotional values. This finding lends support to the hypothesis that children internalise spiritual values in their entirety, as evidenced by the single-factor structure of the MTS-IW.

As posited in numerous studies in the extant literature (see Reimer et al., 2022; Jerome & Kisby, 2019), childhood represents a critical juncture for spiritual development. It is asserted that the values acquired during this period have a formative influence on the lifelong behaviours and attitudes of the individual. In this context, the developed MDS-IQ has the capacity to meaningfully measure prosocial behaviours such as empathy, compassion, truthfulness and sense of justice exhibited by primary school children in their social relationships.

The results of the confirmatory factor analysis (CFA) indicated that the scale is consistent with the conceptual foundations and psychometric requirements. The fit indices, such as the Root Mean Square Error of Approximation (RMSEA) and the Comparative Fit Index (CFI), exceed the limits that are considered acceptable within the relevant literature (Hair et al., 2020). This finding demonstrates that the model possesses adequate structural suitability to assess children's spiritual value levels. The fact that all of the scale items have factor loadings above 0.50 serves to reinforce the measurement power and conceptual consistency.

However, although the majority of scales developed in the context of Turkey in recent years include a religious-based approach, there is a paucity of studies that combine universal, cultural and secular dimensions. At this juncture, the seminal studies by Okan and Ören (2021) and Okan and Şahin (2024) provide foundational insights into the role of religious cooperation in coping with negative emotions during the pandemic. The former study examined the role of religious cooperation in coping with negative emotions during the process of the pandemic, while the latter focused on the development of a scale to measure spiritual coping skills after the crisis. The Spiritual Contradiction Scale, developed by Okan et al. (2024), offers a contemporary example of measuring different dimensions of spirituality at individual and cultural levels.

Furthermore, Chen and Son’s (2024) study demonstrated that spiritual values are not only associated with religious rituals but also with an individual's psychological well-being, inner balance, and resilience. In this respect, the significant correlations obtained between the MDS-SQ and Spiritual Robustness scale support the hypothesis that children's spiritual orientations are related to their psychological resilience levels.

In contrast, Mata-McMahon et al. (2025) emphasised that spirituality in early childhood is characterised by a holistic structure in terms of essence, origin, and action. This perspective is consistent with the notion that the MLS-IW addresses values such as empathy, truthfulness, and responsibility that children exhibit in their social relationships.

As evidenced by the findings of these studies, spiritual structures are evaluated not only in terms of religious rituals but also in a broader psychosocial framework, encompassing value-oriented behaviour, inner balance, social responsibility and the search for meaning (Okan & Ekşi, 2025; Okan et al., 2024). In this context, the MTS-IQ has become a tool that can evaluate children's spiritual structures. These spiritual structures are shaped within the framework of moral dilemmas, social relationships and value orientations that children encounter in their daily lives. These can be considered on a secular and universal value axis.

In conclusion, this research is important not only in terms of the scale development process, but also in terms of providing a different perspective to spiritual value research for children in Turkey. The developed scale provides a scientifically grounded holistic model based on cultural and developmental contexts that extend beyond traditional approaches rooted in religious foundations.

### Contribution of the Research and Recommendations

This research provides the following contributions to the fields of educational sciences, value education, psychological counselling and child development:

- **A new measurement tool was developed:** In the Turkish context, a valid and reliable scale for measuring children's spiritual values at primary school level was developed for the first time in this study. This scale can be used both in academic studies and educational applications.
- **It is functional for educational applications:** Teachers, school counselling services and school administrations can follow the value development of their students through this scale, and classroom activities can be planned accordingly.
- **It can be integrated with psychological counselling practices:** It can be used as a supportive measurement tool in the process of teaching themes such as self-awareness, empathy and responsibility to children in guidance and psychological counselling services.
- **It provides data for policy development processes:** The data obtained from the scale can be guiding in determining educational policies and restructuring values education curricula.

## Suggestions for future studies:

- The scale can be retested by adapting it to different age groups (e.g. middle school, high school).
- Its cultural validity can be tested by applying it in different cultural contexts (e.g. rural–urban differences, migrant children).
- In longitudinal studies, this scale can be used to follow the spiritual development processes of children over time.
- The use of the scale as a tool to evaluate the effectiveness of in-class character education programmes can be investigated.

### Limitations of the Study

Whilst the present study makes a valuable contribution to the existing literature by developing a spiritual values scale for primary school children in Turkey, it is important to note that the study is not without its limitations. Firstly, the sample was limited to a specific age group (primary school children), which restricts the generalizability of the findings to other age levels. Secondly, the scale's applicability was tested only within the Turkish cultural context; thus, further exploration is required to ascertain its cross-cultural validity. Thirdly, the data were collected through self-reports, which may be influenced by social desirability bias, especially when assessing spiritual or moral values. In conclusion, the present study adopted a cross-sectional research design. To comprehensively assess the evolution of children's spiritual values over time, longitudinal research is imperative.

## Data Availability

The datasets generated during and/or analyzed during the current study are available from the corresponding author on reasonable request.
